# Supplementary material for: Association of gender and metabolic factors with thyroid nodules in T2DM: a retrospective study
Source: PeerJ. 2025 Mar 26;13:e19068. doi: 10.7717/peerj.19068 (PMC11954462; doi:10.7717/peerj.19068)
Supplement: Supplemental Information 4 [file peerj-13-19068-s004.docx]

**Table 1** Univariate analyses of factors associated with TNs risk in type 2 diabetic males (n = 474).

| **Variables** | **Total(N)** | **Univariate analysis** | |
| --- | --- | --- | --- |
|  |  | **Odds Ratio (95% CI)** | ***P* value** |
| Age (years) | 474 | 1.039 (1.021 - 1.057) | **< 0.001** |
| DM course (years) | 474 | 1.010 (0.982 - 1.040) | 0.480 |
| Smoking (%) | 239(474) | 1.019 (0.682 - 1.522) | 0.928 |
| Drinking (%) | 345(474) | 1.237 (0.779 - 1.964) | 0.367 |
| ASCVD (%) | 323(474) | 0.687 (0.451 - 1.048) | 0.081 |
| SBP(mmHg) | 474 | 1.004 (0.991 - 1.017) | 0.513 |
| DBP(mmHg) | 474 | 0.993 (0.972 - 1.014) | 0.495 |
| Metformin | 283(474) | 1.315 (0.868 - 1.994) | 0.197 |
| Insulin | 220(474) | 1.273 (0.852 - 1.904) | 0.239 |
| HbA1c (%) | 474 | 0.894 (0.811 - 0.985) | **0.023** |
| UA (μmol/L) | 474 | 1.000 (0.998 - 1.002) | 0.851 |
| TC (mmol/L) | 474 | 0.860 (0.728 - 1.017) | 0.078 |
| TG (mmol/L) | 474 | 0.772 (0.656 - 0.907) | **0.002** |
| HDLC (mmol/L) | 474 | 0.813 (0.472 - 1.400) | 0.456 |
| LDLC (mmol/L) | 474 | 0.854 (0.688 - 1.060) | 0.151 |
| TSH (uIU/ml) | 474 | 0.898 (0.766 - 1.054) | 0.188 |
| Weight (Kg) | 474 | 1.012 (0.994 - 1.030) | 0.211 |
| BMI (kg/m^2^) | 474 | 1.051 (0.989 - 1.117) | 0.108 |
| PBF (%) | 474 | 1.022 (0.989 - 1.056) | 0.187 |
| WHR | 474 | 0.555 (0.027 - 11.390) | 0.702 |
| SLM (Kg) | 474 | 1.008 (0.974 - 1.043) | 0.644 |
| FFM (Kg) | 474 | 1.008 (0.976 - 1.040) | 0.640 |
| SMM (Kg) | 474 | 1.012 (0.960 - 1.067) | 0.654 |
| VFA (cm^3^) | 474 | 1.004 (0.998 - 1.010) | 0.165 |
| AC (cm) | 474 | 1.026 (0.955 - 1.103) | 0.479 |
| Overweight (%) | 330(474) | 1.367 (0.870 - 2.146) | 0.175 |
| Obesity (%) | 127 (474) | 1.274 (0.818 - 1.986) | 0.284 |

**Table 2** Univariate analyses of factors associated with TNs risk in type 2 diabetic females (n = 273).

| **Variables** | **Total(N)** | **Univariate analysis** | |
| --- | --- | --- | --- |
|  |  | **Odds Ratio (95% CI)** | ***P* value** |
| Age (years) | 273 | 1.051 (1.028 - 1.074) | **< 0.001** |
| DM course (years) | 273 | 1.037 (1.002 - 1.073) | **0.038** |
| Smoking | 6 (273) | 1.814 (0.327 - 10.074) | 0.496 |
| ASCVD(%) | 110 (273) | 1.536 (0.942 - 2.504) | 0.085 |
| SBP(mmHg) | 273 | 1.021 (1.006 - 1.037) | **0.007** |
| DBP(mmHg) | 273 | 1.017 (0.993 - 1.042) | 0.171 |
| Metformin | 159 (273) | 1.449 (0.894 - 2.350) | 0.132 |
| Insulin | 132 (273) | 0.761 (0.473 - 1.226) | 0.262 |
| HbA1c (%) | 273 | 0.920 (0.820 - 1.033) | 0.157 |
| UA (μmol/L) | 273 | 1.003 (1.001 - 1.006) | **0.016** |
| TC (mmol/L) | 273 | 0.981 (0.796 - 1.210) | 0.858 |
| TG (mmol/L) | 273 | 1.010 (0.886 - 1.152) | 0.879 |
| HDLC (mmol/L) | 273 | 1.066 (0.477 - 2.381) | 0.876 |
| LDLC (mmol/L) | 273 | 0.992 (0.759 - 1.297) | 0.953 |
| TSH (uIU/ml) | 273 | 1.010 (0.865 - 1.178) | 0.904 |
| Weight (Kg) | 273 | 1.032 (1.007 - 1.059) | **0.014** |
| BMI (kg/m^2^) | 273 | 1.096 (1.023 - 1.175) | **0.010** |
| PBF (%) | 273 | 1.051 (1.011 - 1.093) | **0.012** |
| WHR | 273 | 1.072 (1.029 - 1.118) | **0.001** |
| SLM (Kg) | 273 | 1.030 (0.973 - 1.090) | 0.306 |
| FFM (Kg) | 273 | 1.029 (0.975 - 1.085) | 0.297 |
| SMM (Kg) | 273 | 1.037 (0.949 - 1.134) | 0.424 |
| VFA (cm^3^) | 273 | 1.010 (1.004 - 1.016) | **0.001** |
| AC (cm) | 273 | 1.116 (1.023 - 1.217) | **0.014** |
| Overweight(%) | 169 (273) | 1.534 (0.939 - 2.507) | 0.088 |
| Obesity(%) | 60 (273) | 2.305 (1.256 - 4.227) | **0.007** |

Note: Signifificant associations are highlighted in bold

**Table 3** Characteristics of patients with TNs according to the maximal diameter of the largest thyroid nodule (≥1cm vs <1cm) in type 2 diabetic females.

| **Variables** | **<1cm**  **n=106** | **≥1cm**  **n=37** | **P value** |
| --- | --- | --- | --- |
| Metformin (%) | 68 (64.2%) | 21 (56.8%) | 0.424 |
| TSH (uIU/ml) | 2.085 (1.2725, 3.29) | 2.36 (1.61, 3.41) | 0.446 |

**Table 4** Characteristics of patients with type 2 diabetes mellitus according to the presence of thyroid nodules

|  | **Males** | | |  |  | **Females** | |  |
| --- | --- | --- | --- | --- | --- | --- | --- | --- |
| **Variables** | **Unpresent**  **n=342** |  | **Present**  **n=132** | ***P* value** |  | **Unpresent**  **n=129** | **Present**  **n=144** | ***P* value** |
| FT3 | 2.97 ± 0.35 | | 2.96 ± 0.33 | 0.627 |  | 2.7816 ± 0.30848 | 2.8295 ± 0.31938 | 0.210 |
| FT4 | 12.78 ± 1.33 | | 12.71 ± 1.37 | 0.575 |  | 12.5 (11.6, 13.7) | 12.8 (11.88, 13.8) | 0.165 |
| TSH | 1.91 (1.17, 3.04) | | 1.84 (1.27, 2.54) | 0.206 |  | 2.24 (1.51, 3.54) | 2.19 (1.29, 3.30) | 0.856 |
